# Supplementary material for: In situ dissection of domain boundaries affect genome topology and gene transcription in Drosophila
Source: Nat Commun. 2020 Feb 14;11:894. doi: 10.1038/s41467-020-14651-z (PMC7021724; doi:10.1038/s41467-020-14651-z)
Supplement: Supplementary file 2 — Supplementary Information [file 41467_2020_14651_MOESM2_ESM.pdf]

Supplementary Information

"*In situ* dissection of domain boundaries affect genome topology and gene transcription in *Drosophila*"

Arzate-Mejía et al.

Supplementary Figures

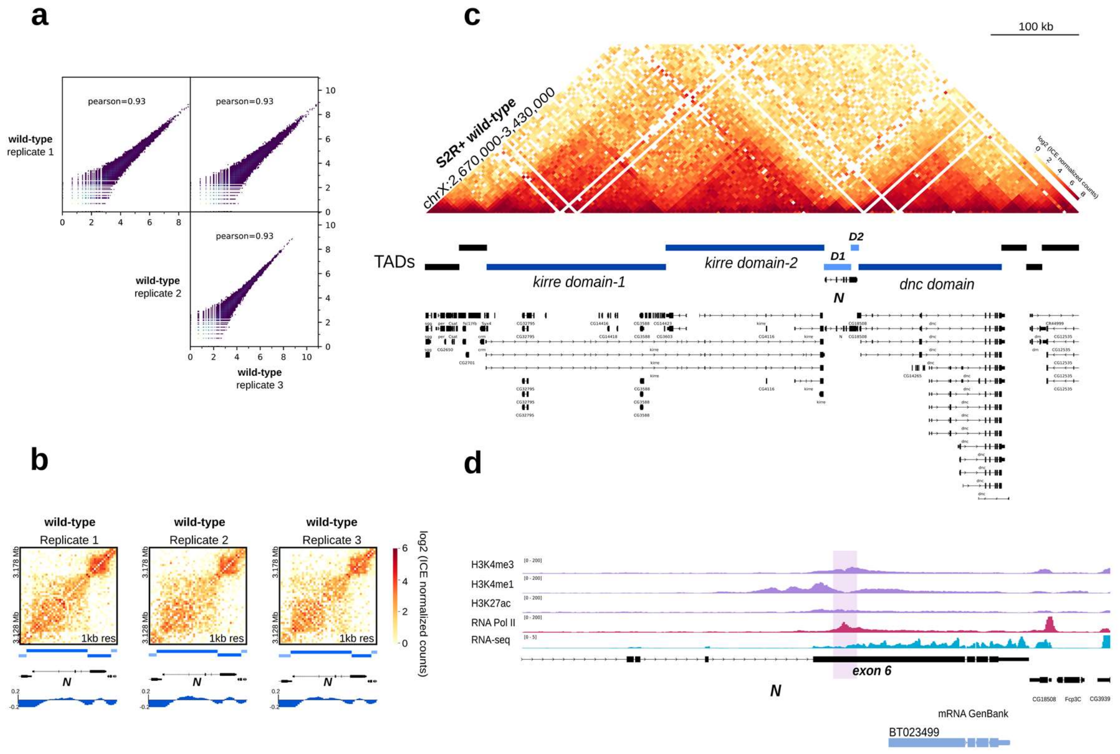

Supplementary Figure 1. Topological domains spanning the *kirre* and *dnc* loci flank *Notch.*

a Scatterplots comparing interactions between wild-type Hi-C replicates. The Pearson correlation value for each pair-wise comparison is shown.

b. Hi-C normalized heatmaps at 1 kb resolution covering a 50 kb region centered in *Notch* for each wild-type replicate. The position of TADs and the TAD separation score for each dataset are shown below each heatmap.

c Hi-C heatmap at 5 kb resolution showing the topological landscape surrounding *Notch*. TADs identified at 1kb resolution are shown below the heatmap. The *kirre* locus is partitioned into two TADs termed *kirre* domain-1 and 2 while the *dnc* locus is fully contained within a TAD.

d Genome browser track displaying part of the *Notch* locus with public ChIP-seq data for histone marks, RNA Pol II and RNA-seq in S2R+ cells. A region enriched with Pol II in exon 6 is highlighted. Below, a cDNA identified for this region and reported in GenBank is shown.

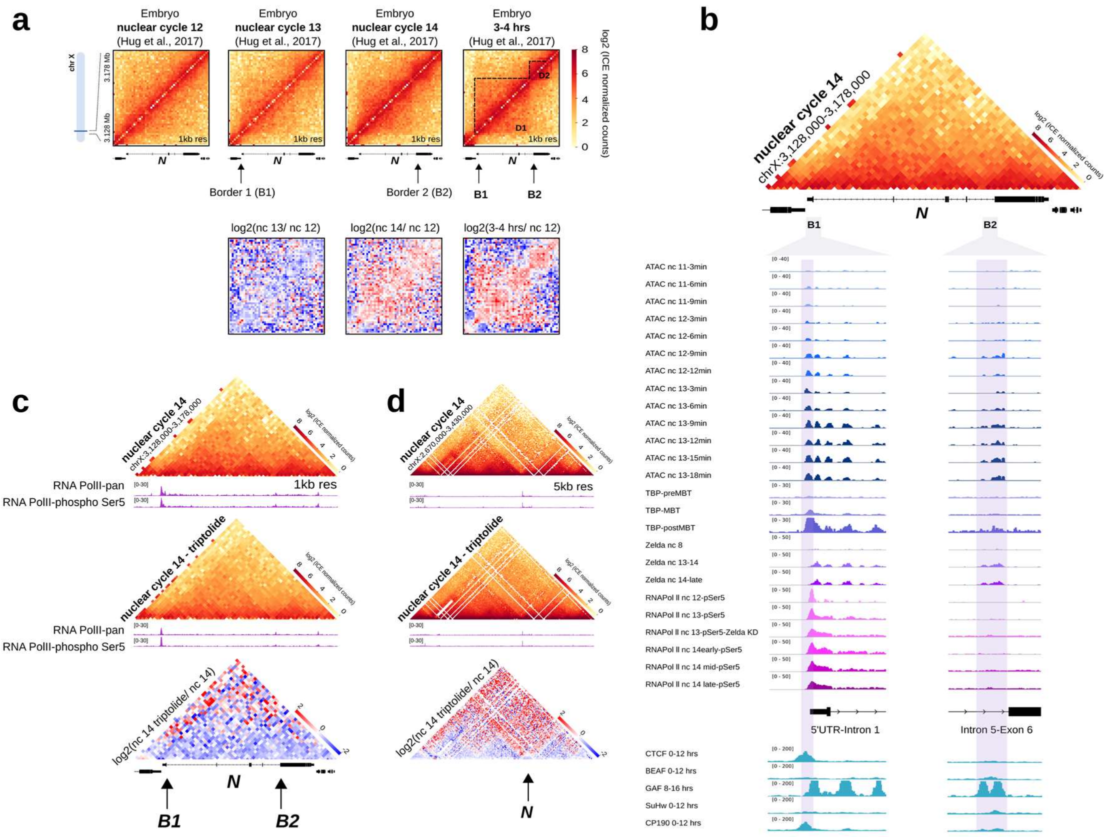

Supplementary Figure 2. *Notch* 3D organization emerges during early embryonic development and correlates with the gain of chromatin accessibility and binding of RNA Pol II at domain boundaries.

a Hi-C heatmaps at 1 kb resolution covering a 50 kb region centered in *Notch*. Hi-C from developing embryos (nuclear cycle 12,13,14 and 3-4 hrs of development; Hug et al., 2017) was re-analyzed using the same pipeline used to analyze the Hi-C data generated in this study. Black arrows indicate the position of B1 and B2 boundaries of *Notch.* Dotted lines indicate the position of the D1 and D2 domains of *Notch*. Below, Hi-C heatmaps of the log2 differences in interaction frequency between the nuclear cycle 12 (nc 12) and nc13, nc14, and 3-4 hrs.

b Triangular representation of a Hi-C heatmap from nuclear cycle 14 wild-type embryos at 1 kb resolution covering a 50 kb region centered in *Notch.* Below the heatmap are shown tracks for public ATAC-seq, and ChIP-seq datasets for TBP, RNA Pol II, and Zelda at different time points during early embryonic development (nc11-nc14) for the regions identified as boundaries at the *Notch* locus. ChIP-seq data for Architectural Proteins from 0-

12 hrs embryos is also shown. MBT, mid blastula transition. Note that the B1 domain boundary is highly accessible and shows a strong enrichment or RNA pol II and TBP.

c Triangular representation of Hi-C heatmaps at 1 kb resolution covering a 50 kb region centered in *Notch*. *Top* and *middle*, Hi-C from nuclear cycle 14 wild-type or triptolide treated embryos. Below each heatmap are shown RNA Pol II ChIP-seq tracks from wild-type, and triptolide treated embryos from Hug et al., 2017. *Bottom*, Hi-C heatmaps of the log2 difference in interaction frequency between wild-type nuclear cycle 14 and nuclear cycle 14 triptolide treated embryos. Black arrows indicate the position of the B1 and B2 boundaries of *Notch.* Observe that global transcriptional inhibition results in a decrease of intradomain interactions at the *Notch* locus; however, TADs are still visible.

d Triangular representation of Hi-C heatmaps at 5 kb resolution covering a 760 kb region centered in *Notch. Top* and *middle*, Hi-C from nuclear cycle 14 wild-type or triptolide treated embryos. Below each heatmap are shown RNA Pol II ChIP-seq tracks from wild-type, and triptolide treated embryos from Hug et al., 2017. *Bottom*, Hi-C heatmaps of the log2 difference in interaction frequency between wild-type nuclear cycle 14 and nuclear cycle 14 triptolide treated embryos. Black arrow indicates the position of *Notch.* Observe that global transcriptional inhibition results in a decrease of intradomain interactions also at TADs flanking the *Notch* locus; however, TADs are still visible.

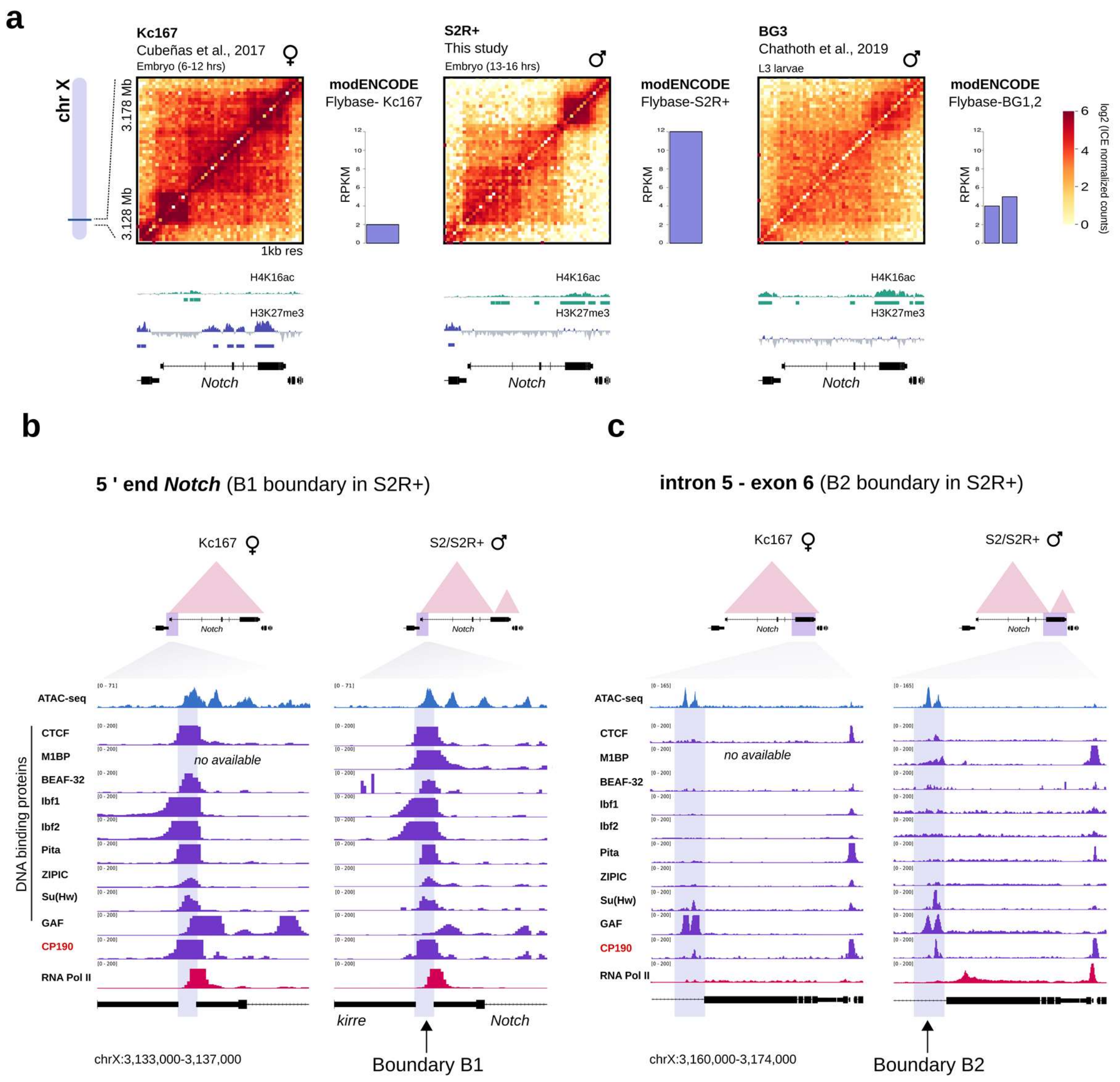

Supplementary Figure 3. The 3D organization of the *Notch* locus in *D. melanogaster* is different between female and male cells and topological differences correlate with the enrichment of H4K16ac and the binding of Architectural Proteins.

a Hi-C normalized heatmaps at 1 kb resolution covering a 50 kb region centered in *Notch* for different *D. melanogaster* cell lines. Public Hi-C data were re-analyzed using the same pipeline used to analyze the Hi-C data generated in this study (see Methods). *Left*, Hi-C heatmap from the embryonic female K167 cell line[1]. *Center*, Hi-C heatmap from the embryonic male S2R+ cell line (this study). *Right*, Hi-C heatmap from the L3 male BG3 cell line[2]. Next to each heatmap the expression level of *Notch* for each cell line is shown as obtained from modENCODE[3]. Below each heatmap ChIP-chip tracks for the histone post-

translational marks H4K16ac and H3K27me3 as obtained from modENCODE[3] are shown. Observe that the two-domain organization of *Notch* in male derived cell lines (S2R+ and BG3) correlates with the enrichment of H4K16ac at the genomic region encompassing the Domain 2.

b Chromatin accessibility and binding profile of different Architectural Proteins and RNA Pol II at the 5' end of *Notch* in Kc167 and S2/S2R+ cells[1,4,5]. On top of ATAC-seq and ChIP-seq tracks is shown a schematic representation of the *Notch* 3D organization in Kc167 and S2R+ cells. Highlighted is the genomic region detected as the B1 boundary of *Notch* in S2R+ cells (this study).

c Chromatin accessibility and binding profile of different Architectural Proteins and RNA Pol II at the genomic region encompassing intron 5-exon 6 of *Notch* in Kc167 and S2/S2R+ cells[1,4,5]. On top of ATAC-seq and ChIP-seq tracks is shown a schematic representation of the *Notch* 3D organization in Kc167 and S2R+ cells. Highlighted is the genomic region detected as the B2 boundary of *Notch* in S2R+ cells (this study). Observe that the presence of the Boundary B2 in S2R+ cells correlate with the binding of CP190 and M1BP at S2/S2R+ cells.

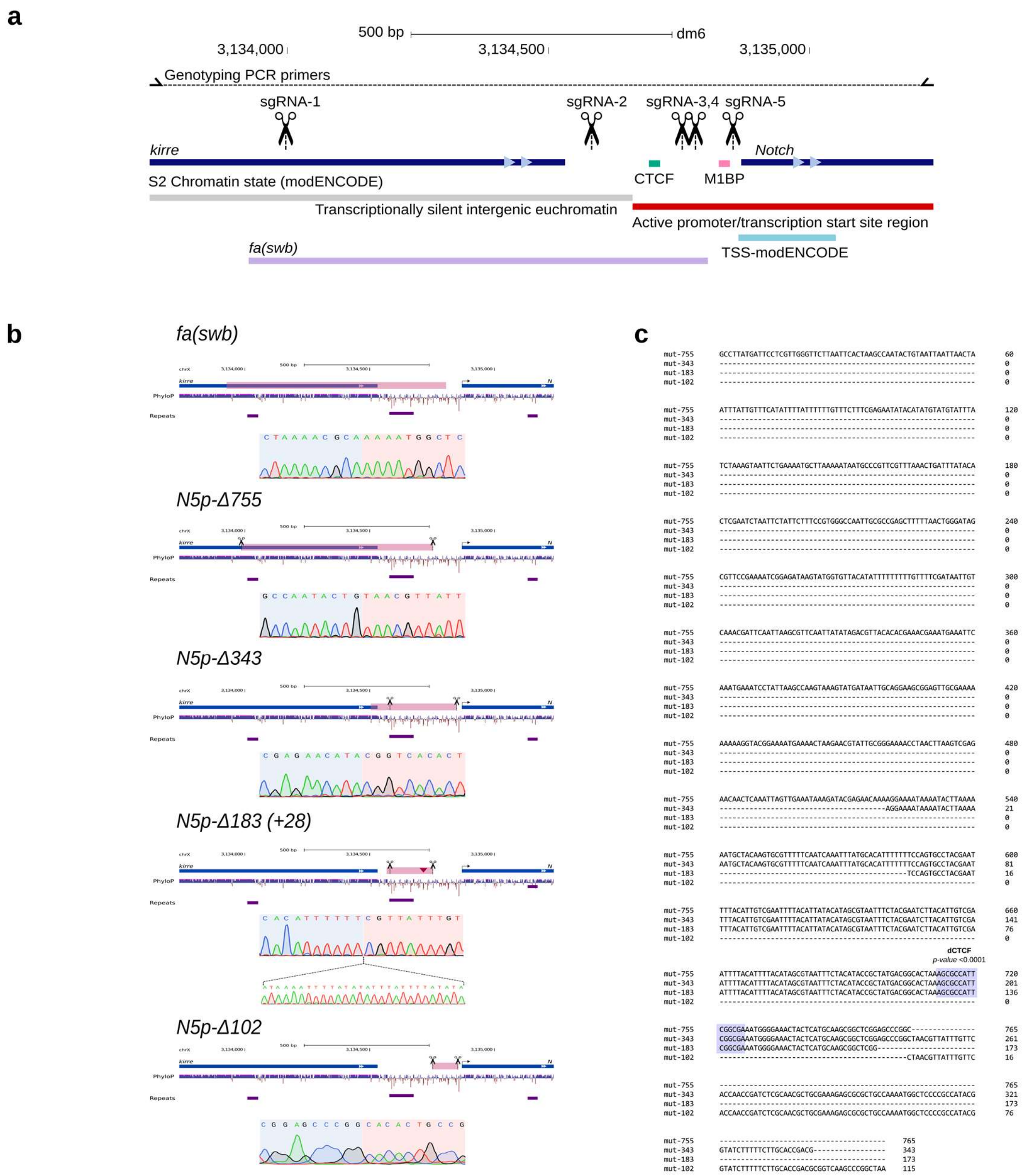

Supplementary Figure 4. CRISPR-Cas9 strategy for deletion of the B1 boundary and sequencing results for mutant clones.

a Design for CRISPR/Cas9 mediated deletions over the 5' intergenic region of *Notch*.

b Sanger-sequencing breakpoints for all mutant alleles generated by CRISPR-Cas9 in S2R+ cells as well as the breakpoints of the *fa(swb)* mutant flies. Pink boxes represent the deleted

sequence in each mutant. Scissors represent the sgRNAs used to generate each deletion. For each mutant an electropherogram of the sequencing results at the deletion breakpoints is shown. A red triangle represents an insertion of 28 bp in the *N5p-Δ183* mutant allele.

c Multiple alignment of the deleted sequences from each 5' mutant generated in this study. Note that with exception of the *N5p- Δ102* allele, all other mutant alleles have lost the binding site for CTCF.

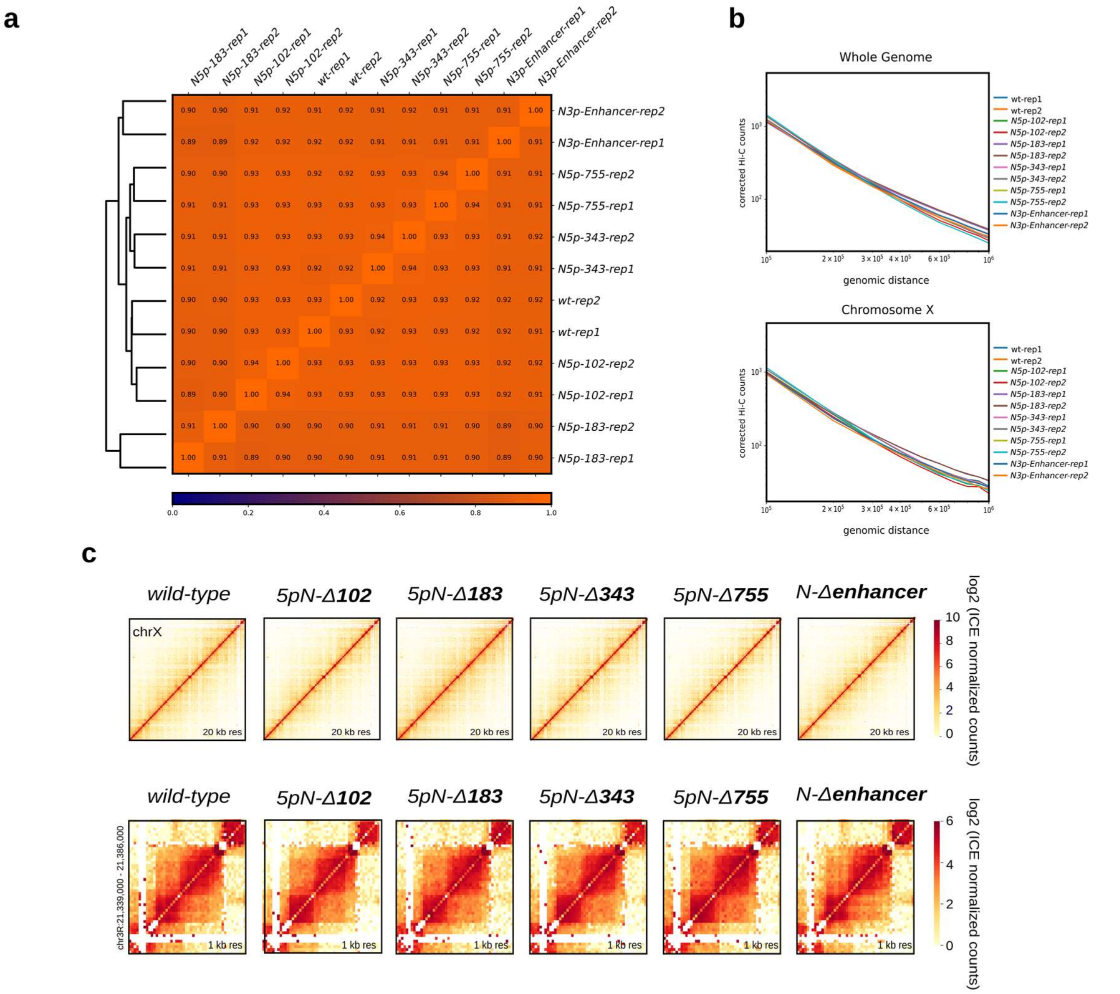

Supplementary Figure 5. Quality Control for Hi-C data from CRISPR mutant clones.

a Pearson correlation heatmap between all Hi-C datasets generated in this study.

b Distance vs HiC counts plots for all Hi-C data sets generated in this study. *Top*, whole-genome. *Bottom*, X chromosome.

c *Top*, Hi-C heatmaps at 20kb resolution for the X chromosome for wild-type and all CRISPR mutants generated in this study. *Bottom*, Hi-C heatmaps at 1 kb resolution centered in *mod(mdg4)* for wild-type and all CRISPR mutants. Observe that *mod(mdg4)* is organized into a TAD with additional subTADs spanning the locus. CRISPR deletion at *Notch* domain boundaries on the X chromosome do not affect the overall organization of the X chromosome nor the organization of a locus in a different chromosome.

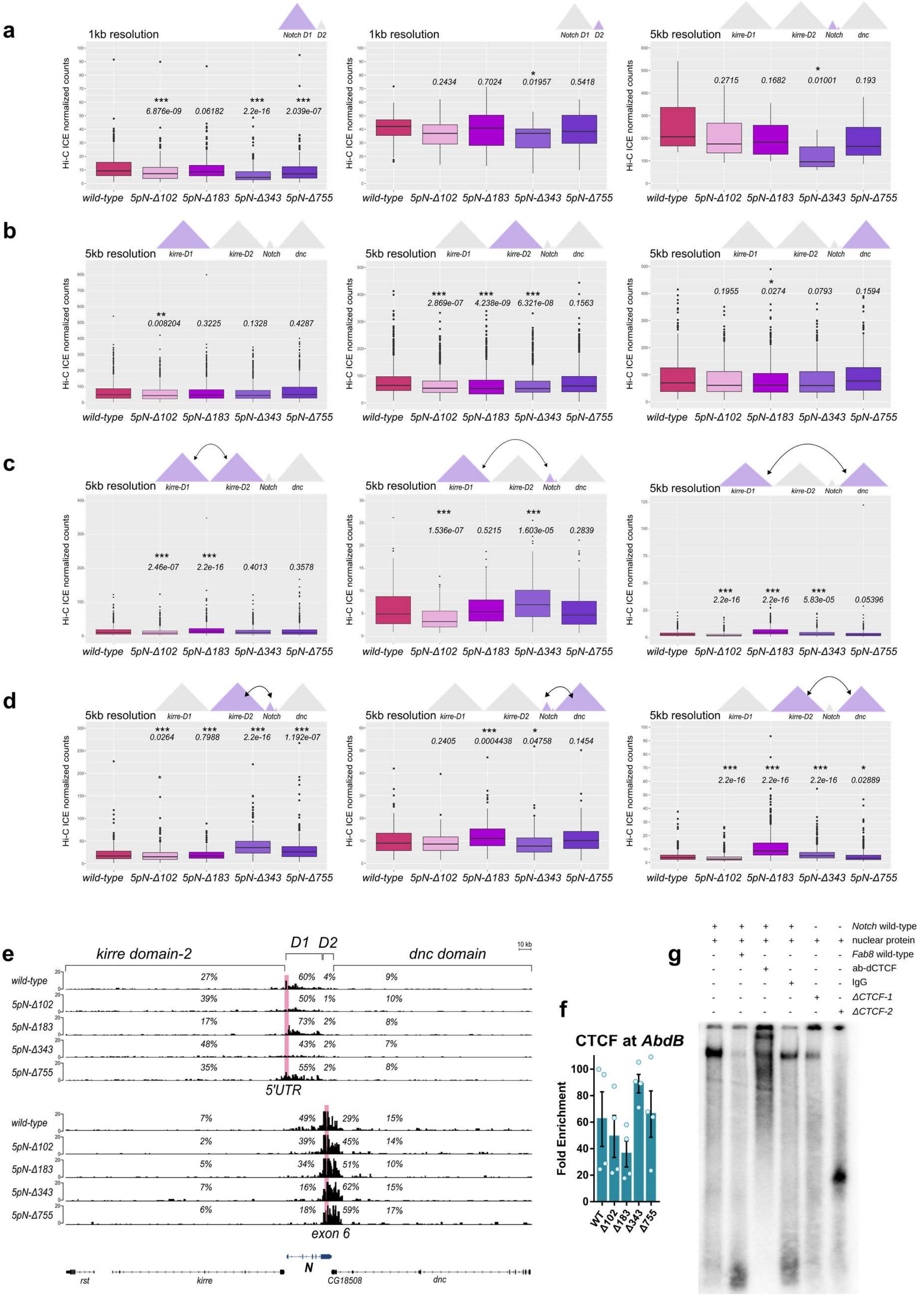
a
b
c
d
e
f
g
1kb resolution
5kb resolution
Notch D1 D2
kirre-D1 kirre-D2 Notch dnc
Hi-C ICE normalized counts
wild-type 5pN-Δ102 5pN-Δ183 5pN-Δ343 5pN-Δ755
6.876e-09 0.06182 2.2e-16 2.039e-07
0.2434 0.7024 0.01957 0.5418
0.2715 0.1682 0.01001 0.193
0.008204 0.3225 0.1328 0.4287
2.869e-07 4.238e-09 6.321e-08 0.1563
0.1955 0.0274 0.0793 0.1594
2.46e-07 2.2e-16 0.4013 0.3578
1.536e-07 0.5215 1.603e-05 0.2839
2.2e-16 2.2e-16 5.83e-05 0.05396
0.0264 0.7988 2.2e-16 1.192e-07
0.2405 0.0004438 0.04758 0.1454
2.2e-16 2.2e-16 2.2e-16 0.02889
kirre domain-2
D1 D2
dnc domain
10 kb
5'UTR
exon 6
rst kirre N CG18508 dnc
CTCF at AbdB
Fold Enrichment
WT Δ102 Δ183 Δ343 Δ755
Notch wild-type
nuclear protein
Fab8 wild-type
ab-dCTCF
IgG
ΔCTCF-1
ΔCTCF-2

Supplementary Figure 6. Deletion of the B1 boundary of *Notch* results in topological defects.

a Boxplots of Hi-C normalized counts for *Notch D1*-1 kb resolution (left), *Notch D2*-1 kb resolution (center) and *Notch* - 5kb resolution (right).

b Boxplots of Hi-C normalized counts for domain *kirre D1* (left), domain *kirre D2* (center) and *dnc* (right) all at 5kb resolution.

c Boxplots of Hi-C normalized counts for inter-domain interactions between domain *kirre D1-kirre D2* (left), domain *kirre D1-Notch* (center) and *kirre D1-dnc* (right) all at 5kb resolution.

d Boxplots of Hi-C normalized counts for inter-domain interactions between domain *kirre D2-Notch* (left), domain *Notch-dnc* (center) and *kirre D2-dnc* (right) all at 5kb resolution.

On top of each plot a diagram depicting the location of the evaluated interactions is shown. *p-values* from a Wilcoxon-Rank Sum Test are shown on top of each boxplot. *p-value* *<0.05, **<0.01,***<0.001. All source data are provided as Source Data File.

e Virtual 4C of Hi-C data using the 5'UTR and the exon 6 of *Notch* as viewpoints for the wild-type and B1 boundary CRISPR mutants. Percentages in each track indicates de fraction of valid-reads interacting with the viewpoint in each domain.

f qChIP against CTCF using a set of primers for the control region *AbdB* for wild-type and CRISSPR mutants. Shown are fold enrichment values over IgG. Error bars represent the Standard Error of the Mean of four replicates (n=4). Source data are provided as Source Data File.

g EMSA using S2R+ protein nuclear extracts and oligonucleotides listed in Table 2. As a control, a non-labeled 60 bp oligonucleotide with a well-validated CTCF binding motif from the *Fab 8* insulator was used. From left to right; 1, shift with the *Notch* wild-type oligonucleotide; 2, competition of the labeled *Notch* wild-type oligonucleotide with the non-labeled *Fab8* oligonucleotide; 3, super-shift assay with protein nuclear extracts incubated with α-dCTCF and the *Notch* wild-type oligonucleotide; 4, incubation of wild-type oligonucleotide with IgG; 5 and 6 CTCF mutant oligonucleotides incubated with protein nuclear extracts. Note that mutant oligonucleotides either reduce the binding of nuclear proteins (ΔCTCF-1) or completely disrupt wild-type shift (ΔCTCF-2). Source data are provided as Source Data File.

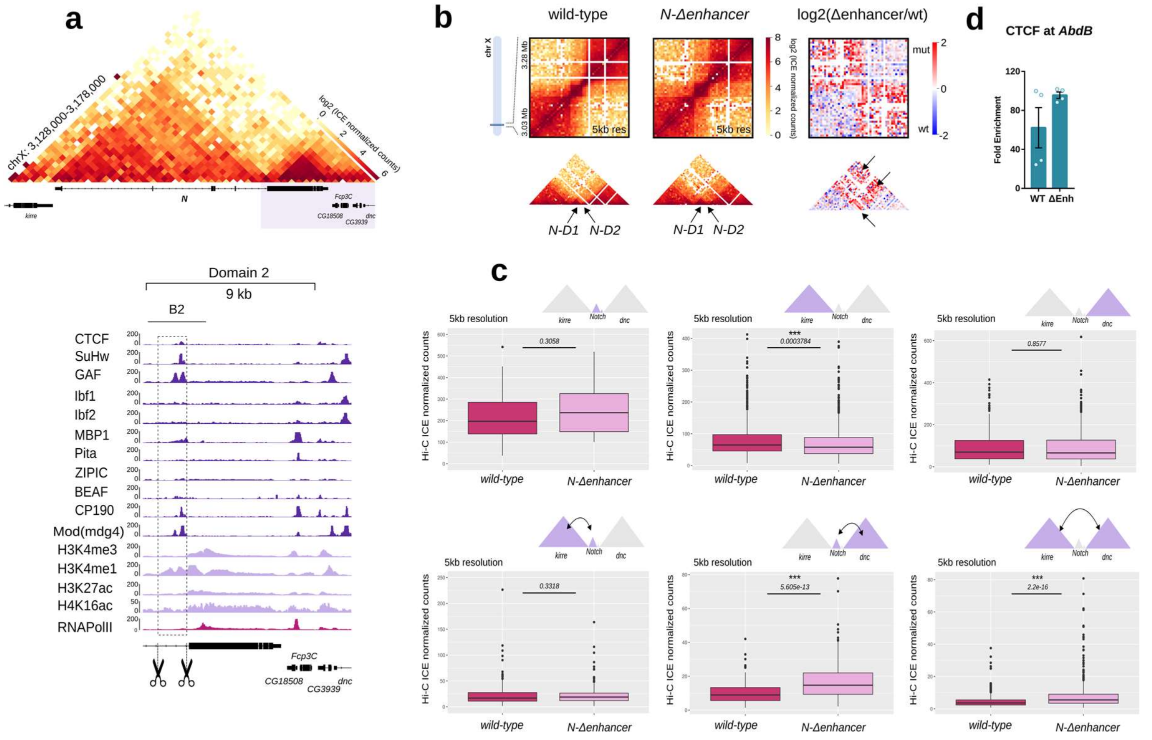

Supplementary Figure 7. Deletion of the B2 boundary of *Notch* results in local and long-range topological defects.

a The B2 boundary of *Notch* overlaps an intronic enhancer occupied by CP190 and other Architectural Proteins (APs). A triangular representation of a Hi-C heatmap at 1 kb resolution covering a 50 kb region and centered in *Notch* is shown on top. Highlighted is a region covering from the B2 boundary to the end of the D2 domain of *Notch*. This genomic region is shown below with ChIP-seq tracks for APs, histone marks, and RNA Pol II. Scissors represent the position of sgRNAs used for CRISPR-Cas9 mediated deletion of the intronic enhancer.

b Deletion of the intronic enhancer results in increased inter-domain interactions between TADs flanking *Notch*. *Left* and *center*, heatmaps at 5 kb resolution of Hi-C data for wild-type and the enhancer mutant covering a region of ~250 kb centered in *Notch*. *Right*, heatmap of the log2 differences between wild-type and the enhancer mutant. The arrows in highlight regions that show a gain of interactions in the enhancer mutant.

c Boxplots of Hi-C normalized counts for *kirre*-domain 1 and 2, *Notch* domains and *dnc*-domain as well as inter-domain interactions in wild-type and the enhancer mutants. On top of each plot, a diagram depicting the location of the evaluated interactions is shown. *p-values* from a Wilcoxon-Rank Sum Test are shown on top of each boxplot. *p-value* *<0.05, **<0.01,***<0.001. Source data are provided as Source Data File.

d qChIP against CTCF using a set of primers for the control region *AbdB* for wild-type and enhancer mutants. Fold enrichment over IgG. Error bars represent the Standard Error of the Mean of four replicates (n=4). Source data are provided as Source Data File. Source data are provided as Source Data File.

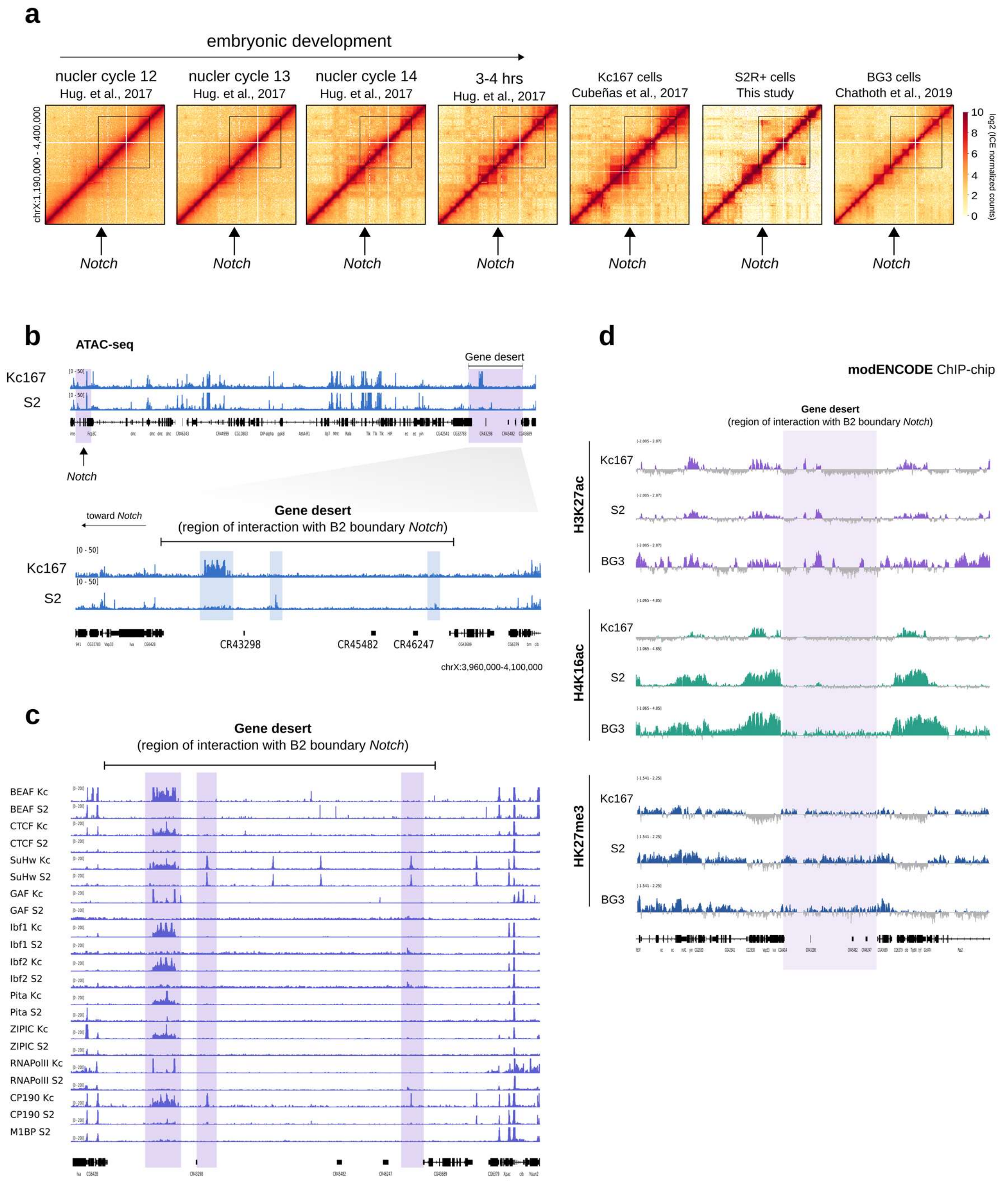

Supplementary Figure 8. The *Notch* megadomain is unique to S2R+ cells and its anchors show differential chromatin accessibility, binding of Architectural Proteins and histone marks between different cell types.

a Hi-C normalized heatmaps at 20 kb resolution for different stages of *D. melanogaster* embryonic development and cell lines. A black square in each heatmap shows the region encompassing the *Notch* megadomain detected in S2R+ cells.

b ATAC-seq profile derived from public datasets of the *Notch* megadomain (top) and the gene desert region (bottom) in Kc167 and S2 cells[4]. Observe that the chromatin accessibility profile for both cell lines is different at the gene desert region (blue rectangles highlight regions with differential accessibility).

c ChIP-seq tracks derived from public datasets for Architectural Proteins (APs) at the gene desert in Kc and S2 cells[1,5]. Observe that the binding of APs for both cell lines is different at the gene desert region (purple rectangles highlight regions with differential binding of APs).

d ChIP-chip tracks derived from modENCODE for histone post-translational modifications at the gene desert[3]. Observe that the profile of enrichment for histone post-translational modifications is different at the gene desert region between cell lines (purple rectangles highlight regions with enrichment for histone post-translational modifications).

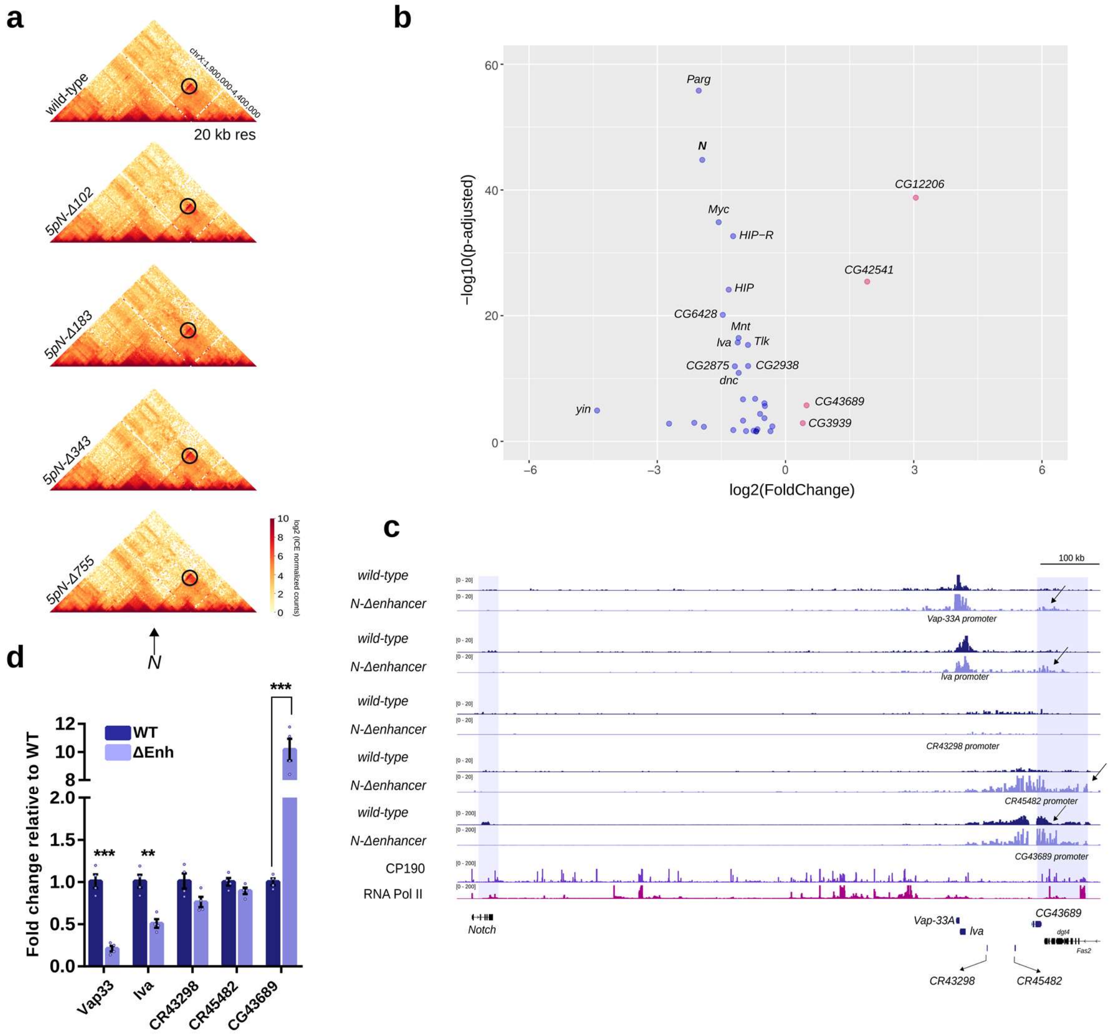

Supplementary Figure 9. Deletion of the B2 boundary in S2R+ cells eliminates the *N*^{enh}-mega-domain and affect transcription of the genes located within the mega-domain.

a Triangular representation of Hi-C heatmaps covering a 2.5 Mb region at 20 kb resolution for wild-type cells and the B1 boundary CRISPR mutants centered in *Notch*. Highlighted by a black circle is a 1 Mb long-range interaction mediated by the intronic enhancer of *Notch*. Observe that disruption of the B1 boundary does not affect the *N*^{enh}-mega-domain.

b Volcano plot of the differentially expressed genes within the *N*^{enh}-mega-domain between the wild-type and enhancer mutant cells. Blue dots represent genes downregulated in the enhancer mutant while red dots represent genes upregulated in the enhancer mutant cells.

c Virtual 4C of Hi-C data using the promoter of genes located at or near the gene desert as viewpoints for the wild-type and the enhancer mutant. Arrows indicate regions with ectopic interactions. ChIP-seq tracks for CP190 and RNA Pol II are shown. Highlighted regions correspond to the anchors of the N^{enh}-mega-domain.

d Transcription of genes located at or near the gene desert interacting with the intronic enhancer of *Notch* in wild-type and the enhancer mutant cells. Significant differences between wild-type and enhancer mutant were calculated using a t-test. *p-value* *<0.05, **<0.01,***<0.001. Error bars represent the Standard Error of the Mean of four replicates (n=4). Source data are provided as Source Data File.

Supplementary Tables

	wild-type	*5pN-Δ102*	*5pN-Δ183*	*5pN-Δ343*	*5pN-Δ755*	*Δenhancer*
valid_interaction	73,371,778	80,145,697	65,248,465	74,781,396	68,610,520	68,348,417
valid_interaction_rmdup	72,953,774	79,091,829	64,026,434	73,588,490	67,582,002	67,252,940
trans_interaction	4,426,563	3,114,910	5,925,899	2,640,130	2,462,914	5,104,348
%trans_interaction	6.06762715	3.93834615	9.25539442	3.58769422	3.64433418	7.58977674
cis_interaction	68,527,211	75,976,919	58,100,535	70,948,360	65,119,088	62,148,592
%cis_interaction	93.9323728	96.0616539	90.7446056	96.4123058	96.3556658	92.4102233
cis_shortRange	33,897,767	39,009,711	28,353,246	37,199,484	34,714,319	32,296,687
cis_longRange	34,629,444	36,967,208	29,747,289	33,748,876	30,404,769	29,851,905

Supplementary Table 1. Hi-C statistics. HiC-Pro summary for Hi-C merged datasets generated in this study. Highlighted in blue are the number of valid pairs used in all analysis presented in this manuscript.

Supplementary Table 2

Name	Sequence 5'-3'	Usage	Notes
sgRNA-1	TTATGATTCCTCGTTGGGTT	sgRNA	755-5p
sgRNA-2	GTGCCTACGAATTTTACATT	sgRNA	183-5p and 343-5p
sgRNA-3	ACGCGGTCACACTGCCGATT	sgRNA	102-3p and 343-3p
sgRNA-4	GGCTAACGTTATTTGTTCAC	sgRNA	102-5p, 183-3p and 755-3p
N5p_F	GCATAAATGTGTATGTCAACGCT	genotypification	CRISPR 5p N mutants
N5p_R	CGGCTTTCGTCTCACTCTCA	genotypification	
enh_F	CGCAATTTTCGCCGAGATTT	genotypification	CRISPR enhancer mutants
enh_R	CAGTTCTTGCCCTCGAAACC	genotypification	
AbdB_F	CCGCCCCTTTTCGAATACAG	ChIP	control
AbdB_R	TGCACCCTCCTAGTCTAGTG	ChIP	
5pN_F	AGCGTAATTTCTACATACCGCT	ChIP	ChIP for 5p end of N
5pN_R	TGGTGAACAAATAACGTTAGCC	ChIP	
enh_F	ACTTTGTTTGCCCATTCGCT	ChIP	ChIP for enhancer of N
enh_R	CCAACTGCGGCCACAATTAT	ChIP	
prom_exon6_F	TCAAGTGCCTCTGTGATCCC	ChIP	ChIP for exonic promoter of N
prom_exon6_R	AATAGTAGCTGCCCACACGA	ChIP	
Δ102_F	TACCGCTATGACGGCACTAA	ChIP	ChIP for CRISPR mutant
Δ102_R	GCTTTCACAACCGTTTCGTC	ChIP	
Δ183_F	TGCTACAAGTGCGTTTTTCAA	ChIP	ChIP for CRISPR mutant
Δ183_R	ACAACCGTTTCGTCCACTG	ChIP	
Δ343_F	CGTATTGCGGGAAAACCTAA	ChIP	ChIP for CRISPR mutant
Δ343_R	GCTTTCACAACCGTTTCGTC	ChIP	
Δ755_F	AGAAAAACTTAAAAAGCTTAGAAACAA	ChIP	ChIP for CRISPR mutant
Δ755_R	CGTCGGTGCAAGAAAAAGA	ChIP	
Δenh_F	TCTTATTGCAACGTGATGTCCA	ChIP	ChIP for CRISPR mutant
Δenh_R	GGGTAAAACGGTCACACTTCC	ChIP	
rst_F	TGCACGGTGGTCAACGATTA	RT-PCR	kirre TAD
rst_R	CCAAAATGGTCAGCACCAGC	RT-PCR	

kirre_med_F	GCAATTGAACTGGCCAACGA	RT-PCR	kirre TAD
kirre_med_R	CCAAAGTTTGCTGACCGACG	RT-PCR	
kirre_full_F	TCGCGGTAATTTTGCGTTTGA	RT-PCR	kirre TAD
kirre_full_R	TGGCGGCAACCTCAATTCTA	RT-PCR	
Vap33-A_F	TCGATCAACCCAGGTGGAGA	RT-PCR	N-enhancer-megadomain
Vap33-A_R	GCGGTGGGCATCTCGAAAAC	RT-PCR	
lva_F	GGATATACGCTTAGCGGGCA	RT-PCR	N-enhancer-megadomain
lva_R	TGGACTTGTGTCGCTCATCC	RT-PCR	
CG18508_F	CCGGAGCCAGTAAGCTAACATT	RT-PCR	N-enhancer-megadomain
CG18508_R	ACAGGATCGGCTGCACAAAT	RT-PCR	
CG6428_F	CCAGGATGTCAAAGAGGCCC	RT-PCR	N-enhancer-megadomain
CG6428_R	GGCATTGGGAATGGGAGCTA	RT-PCR	
CR43298_F	AAAGGGTTTGGCGTAGGGTT	RT-PCR	N-enhancer-megadomain
CR43298_R	TGAAGGACATTCACCGGACG	RT-PCR	
CR45482_F	AAGCTAAATGGCCCGTGGAA	RT-PCR	N-enhancer-megadomain
CR45482_R	AAGCCCATTCGAACCGAGAG	RT-PCR	
CG43689_F	CCAGGGCATAAAGAAGCCCA	RT-PCR	N-enhancer-megadomain
CG43689_R	ATTGAGACCAAGTCCCACGC	RT-PCR	
N_exon1_intron1_F	CGGAACTGGTGAGTAAAGCG	RT-PCR	Notch Domain 1
N_exon1_intron1_R	AATTCCAGTTTGTGCACCCA	RT-PCR	
N_exon1-exon2-F	TAACAAAATGCACGCCGTTG	RT-PCR	Notch Domain 1
N_exon1-exon2-R	ATTCTGGCAACCGACACTTG	RT-PCR	
N_exon4-exon5_F	ACACCTGTTCCTATGACATCGA	RT-PCR	Notch Domain 1
N_exon4-exon5_R	TTGGTGTCGCAATCCTTTCC	RT-PCR	
N_exon5-exon6_F	GGAATTTGCCGGTCGAACG	RT-PCR	Notch Domain 1
N_exon5-exon6_R	GGTGTAGTCCGAGATGCCAT	RT-PCR	
N_exon8-exon9_F	AGGCCTGGAGTTCGGTTC	RT-PCR	Notch Domain 2
N_exon8-exon9_R	AGCCGGACATTGAACTTTGT	RT-PCR	
Rp49-F	AGCATACAGGCCCAAGATCG	RT-PCR	Endogenus control
Rp49-R	TGTTGTCGATACCCTTGGGC	RT-PCR	
dnc_F	GGAGCAAAACTCGAGCGGTA	RT-PCR	dnc TAD
dnc_R	CCATTTTCTACATCGAAAGGCGA	RT-PCR	
Fab8_sense	AAATTTCCACATTCCCGCCTTGCAGCGCCACCTGGCCTTGGTAATGTAGAACTAGGAAGG	EMSA	Control for CTCF binding

Fab8_antisense	CCTTCCTAGTTCTACATTACCAAGGCCAGGTGGCGCTGCAAGGCGGGAATGTGGAAATTT	EMSA	
N_ctcf_wt_sense	CATACCGCTATGACGGCACTAAAGCGCCATTCGGCGAAATGGGGAAACTACTCATGCAAG	EMSA	wild-type sequence
N_ctcf_wt_antisense	CTTGCATGAGTAGTTTCCCCATTTCGCCGAATGGCGCTTTAGTGCCGTCATAGCGGTATG	EMSA	
N_ctcf_mut1_sense	CATACCGCTATGACGGCACTAAAGTATTATTTGGCAAAATGGGGAAACTACTCATGCAAG	EMSA	mutant sequence for CTCF
N_ctcf_mut1_antisense	CTTGCATGAGTAGTTTCCCCATTTTGCCAAATAATACTTTAGTGCCGTCATAGCGGTATG	EMSA	
N_ctcf_mut2_sense	CATACCGCTATGACGGCACTAAAGCGCCATTCAATAAAATGGGGAAACTACTCATGCAAG	EMSA	mutant sequence for CTCF
N_ctcf_mut2_antisense	CTTGCATGAGTAGTTTCCCCATTTTATTGAATGGCGCTTTAGTGCCGTCATAGCGGTATG	EMSA	

Supplementary Table 2. sgRNAs, primers and oligonucleotides used in this study.

Supplementary References

1. Cubeñas-Potts, C. *et al.* Different enhancer classes in Drosophila bind distinct architectural proteins and mediate unique chromatin interactions and 3D architecture. *Nucleic Acids Res.* **45**, 1714–1730 (2017).
2. Chathoth, K. T. & Zabet, N. R. Chromatin architecture reorganization during neuronal cell differentiation in Drosophila genome. *Genome Res.* **29**, 613–625 (2019).
3. modENCODE Consortium *et al.* Identification of functional elements and regulatory circuits by Drosophila modENCODE. *Science* **330**, 1787–1797 (2010).
4. Albig, C. *et al.* Factor cooperation for chromosome discrimination in Drosophila. *Nucleic Acids Res.* **47**, 1706–1724 (2019).
5. Li, L. *et al.* Widespread Rearrangement of 3D Chromatin Organization Underlies Polycomb-Mediated Stress-Induced Silencing. *Mol. Cell* **58**, 216–231 (2015).
