# Supplementary material for: In situ dissection of domain boundaries affect genome topology and gene transcription in Drosophila
Source: Nat Commun. 2020 Feb 14;11:894. doi: 10.1038/s41467-020-14651-z (PMC7021724; doi:10.1038/s41467-020-14651-z)
Supplement: Supplementary file 3 — Reporting Summary [file 41467_2020_14651_MOESM3_ESM.pdf]

nature research

Corresponding author(s): Furlan-Magaril Mayra and Recillas-Targa Félix

Last updated by author(s): Jan 6, 2020

Reporting Summary

Nature Research wishes to improve the reproducibility of the work that we publish. This form provides structure for consistency and transparency in reporting. For further information on Nature Research policies, see Authors & Referees and the Editorial Policy Checklist.

Statistics

For all statistical analyses, confirm that the following items are present in the figure legend, table legend, main text, or Methods section.

n/a	Confirmed	
☐	☒	The exact sample size (*n*) for each experimental group/condition, given as a discrete number and unit of measurement
☐	☒	A statement on whether measurements were taken from distinct samples or whether the same sample was measured repeatedly
☐	☒	The statistical test(s) used AND whether they are one- or two-sided<br>*Only common tests should be described solely by name; describe more complex techniques in the Methods section.*
☒	☐	A description of all covariates tested
☒	☐	A description of any assumptions or corrections, such as tests of normality and adjustment for multiple comparisons
☐	☒	A full description of the statistical parameters including central tendency (e.g. means) or other basic estimates (e.g. regression coefficient) AND variation (e.g. standard deviation) or associated estimates of uncertainty (e.g. confidence intervals)
☒	☐	For null hypothesis testing, the test statistic (e.g. *F*, *t*, *r*) with confidence intervals, effect sizes, degrees of freedom and *P* value noted<br>*Give P values as exact values whenever suitable.*
☒	☐	For Bayesian analysis, information on the choice of priors and Markov chain Monte Carlo settings
☒	☐	For hierarchical and complex designs, identification of the appropriate level for tests and full reporting of outcomes
☒	☐	Estimates of effect sizes (e.g. Cohen's *d*, Pearson's *r*), indicating how they were calculated

Our web collection on statistics for biologists contains articles on many of the points above.

Software and code

Policy information about availability of computer code

Data collection	No software was used. All data were either generated experimentally for this study or were publicly available and downloaded. The corresponding accession numbers are specified in the methods section.
Data analysis	Data analysis in this study is based mostly in public available software and listed below:<br><br>Software Version Availability<br>HiC-Pro 2.11.0-beta https://github.com/nservant/HiC-Pro<br>HiCExplorer 3 https://hicexplorer.readthedocs.io/en/latest/content/installation.html<br>HiCcompare 1.6.0 https://bioconductor.org/packages/release/bioc/html/HiCcompare.html<br>HOMER v4.10 http://homer.ucsd.edu/homer/index.html<br>HiC-Plotter 0.8.1 https://github.com/kcakdemir/HiCPlotter<br>Seqmonk v1.42.0 https://www.bioinformatics.babraham.ac.uk/projects/seqmonk/<br>Bowtie2 2.3.0 http://bowtie-bio.sourceforge.net/bowtie2/index.shtml<br>FIMO 4.12.0 http://meme-suite.org/doc/download.html<br>IGV 2.3.93 https://software.broadinstitute.org/software/igv/<br>Deeptools 2.5.4 https://deeptools.readthedocs.io/en/develop/<br>DESeq2 1.24.0 https://bioconductor.org/packages/release/bioc/html/DESeq2.html<br>Salmon 0.10.2 https://combine-lab.github.io/salmon/<br>R 3.4.4 https://cran.r-project.org/<br>R studio 1.1.383 https://www.rstudio.com/products/rstudio/download/<br><br>Public software was used either with default paramethers or with specific options as outlined in the Methods section of the Manuscript.<br><br>Custom code to create in silico genomes for read mapping of Hi-C experiments and for quantification of virtual-4C and to create Hi-C counts tables and blox plots from ICE matrices are provided as a separed txt file.

For manuscripts utilizing custom algorithms or software that are central to the research but not yet described in published literature, software must be made available to editors/reviewers. We strongly encourage code deposition in a community repository (e.g. GitHub). See the Nature Research guidelines for submitting code & software for further information.

Data

Policy information about availability of data

All manuscripts must include a data availability statement. This statement should provide the following information, where applicable:

- Accession codes, unique identifiers, or web links for publicly available datasets
- A list of figures that have associated raw data
- A description of any restrictions on data availability

Raw data for in nucleus Hi-C and RNA-seq experiements generated in this study is available at Gene Expression Omnibus as a SuperSeries under the accession number: GSE136137.

Field-specific reporting

Please select the one below that is the best fit for your research. If you are not sure, read the appropriate sections before making your selection.

☒ Life sciences ☐ Behavioural & social sciences ☐ Ecological, evolutionary & environmental sciences

For a reference copy of the document with all sections, see nature.com/documents/nr-reporting-summary-flat.pdf

Life sciences study design

All studies must disclose on these points even when the disclosure is negative.

Sample size	No sample size calculation was performed
Data exclusions	No data were excluded from the analysis
Replication	All attempts at replication were successful
Randomization	This is not relevant for our study. Samples were classified as WT or mutants as specified in the manuscript.
Blinding	This is not relevant for our study. Samples were classified as WT or mutants as specified in the manuscript.

Reporting for specific materials, systems and methods

We require information from authors about some types of materials, experimental systems and methods used in many studies. Here, indicate whether each material, system or method listed is relevant to your study. If you are not sure if a list item applies to your research, read the appropriate section before selecting a response.

Materials & experimental systems

n/a	Involved in the study	
☐	☒	Antibodies
☐	☒	Eukaryotic cell lines
☒	☐	Palaeontology
☒	☐	Animals and other organisms
☒	☐	Human research participants
☒	☐	Clinical data

Methods

n/a	Involved in the study	
☒	☐	ChIP-seq
☒	☐	Flow cytometry
☒	☐	MRI-based neuroimaging

Antibodies

Antibodies used

anti-H3K4me3 Abcam 8580
anti-H3K27ac Abcam 4729
anti-H3K27me3 Abcam 6002
anti-RNAPolII-pSer2 Abcam 5095
anti-RNAPolII-pSer5 Abcam 5408
anti-IgGmouse-Millipore 12-371
anti-IgGRabbit-Millipore-12-370
anti-dCTCF Custom; Polyclonal antibody generated by New England Peptide by immunizing rabbits with a peptide corresponding

	to the first 20 aminoacids of dCTCF as described in Moon H et al., 2005.
Validation	anti-H3K4me3 Abcam;Rabbit polyclonal to Histone H3 (tri methyl K4); Validadted by WB, IF and ChIP; ChIP Grade; Reacts with Drosophila melanogaster<br>anti-H3K27ac Abcam;Rabbit polyclonal to Histone H3 (acetyl K27); Validadted by WB, IF and ChIP; ChIP Grade; Reacts with Drosophila melanogaster<br>anti-H3K27me3 Abcam;Mouse monoclonal to Histone H3 (tri methyl K27) - ChIP Grade; Validadted by WB, IF and ChIP; ChIP Grade; Reacts with Drosophila melanogaster<br>anti-RNAPolII-pSer2 Abcam;Rabbit polyclonal to RNA polymerase IICTD repeat YSPTSPS (phospho S2); Validadted by WB, IF and ChIP; ChIP Grade; Reacts with Drosophila melanogaster<br>anti-RNAPolII-pSer5 Abcam;Mouse monoclonal to RNA polymerase IICTD repeat YSPTSPS (phospho S5); Validadted by WB, IF and ChIP; ChIP Grade; Reacts with Drosophila melanogaster<br>anti-dCTCF New England Peptide; Validated by WB on S2R+ cells and CTCF-null flies as well as by ChIP against AbdB a validated target for dCTCF.

Eukaryotic cell lines

Policy information about cell lines

Cell line source(s)	Drosophila Genomics Resource Center (DGRC)
Authentication	None of the cell lines used were authenticated as they are from a commercial source
Mycoplasma contamination	None of the cell lines used were authenticated as they are from a commercial source
Commonly misidentified lines (See ICLAC register)	*Name any commonly misidentified cell lines used in the study and provide a rationale for their use.*
